# Children Who Acquire HIV Infection Perinatally Are at Higher Risk of Early Death than Those Acquiring Infection through Breastmilk: A Meta-Analysis

**DOI:** 10.1371/journal.pone.0028510

**Published:** 2012-02-23

**Authors:** Renaud Becquet, Milly Marston, François Dabis, Lawrence H. Moulton, Glenda Gray, Hoosen M. Coovadia, Max Essex, Didier K. Ekouevi, Debra Jackson, Anna Coutsoudis, Charles Kilewo, Valériane Leroy, Stefan Z. Wiktor, Ruth Nduati, Philippe Msellati, Basia Zaba, Peter D. Ghys, Marie-Louise Newell, the UNAIDS Child survival group

**Affiliations:** 1 Institut National de la Santé et de la Recherche Médicale (INSERM), Unité 897, Centre de Recherche “Epidémiologie et Biostatistique”, Bordeaux, France; 2 Institut de Santé Publique Epidémiologie Développement (ISPED), Université Victor Segalen Bordeaux 2, Bordeaux, France; 3 London School of Hygiene and Tropical Medicine, London, United Kingdom; 4 Department of International Health, Johns Hopkins Bloomberg School of Public Health, Baltimore, Maryland, United States of America; 5 Perinatal HIV Research Unit (PHRU), University of the Witwatersrand, Soweto, South Africa; 6 HIV Management, Maternal, Adolescent and Child Unit, University of the Witwatersrand, Soweto, South Africa; 7 Department of Immunology and Infectious Diseases, Harvard School of Public Health AIDS Initiative, Boston, Massachusetts, United States of America; 8 PAC-CI program, ANRS site in Côte d'Ivoire, Abidjan, Côte d'Ivoire; 9 School of Public Health, University of the Western Cape, Cape Town, South Africa; 10 Department of Paediatrics and Child Health, University of KwaZulu-Natal, South Africa; 11 Muhimbili University of Health and Allied Sciences, Dar es Salaam, Tanzania; 12 RETRO-CI project, Centers for Disease Control and Prevention, Global HIV/AIDS Program, Abidjan, Côte d'Ivoire; 13 Department of Pediatrics, University of Nairobi, Nairobi, Kenya; 14 Service de Pédiatrie, CHU Sourô Sanou, Bobo Dioulasso, Burkina Faso; 15 UNAIDS, Epidemiology and Analysis Division, Geneva, Switzerland; 16 Africa Centre for Health and Population Studies, University of KwaZulu-Natal, Somkhele, South Africa; 17 Centre for Paediatric Epidemiology and Biostatistics, University College London, Institute of Child Health, London, United Kingdom; Aga Khan University, Pakistan

## Abstract

**Background:**

Assumptions about survival of HIV-infected children in Africa without antiretroviral therapy need to be updated to inform ongoing UNAIDS modelling of paediatric HIV epidemics among children. Improved estimates of infant survival by timing of HIV-infection (perinatally or postnatally) are thus needed.

**Methodology/Principal Findings:**

A pooled analysis was conducted of individual data of all available intervention cohorts and randomized trials on prevention of HIV mother-to-child transmission in Africa. Studies were right-censored at the time of infant antiretroviral initiation. Overall mortality rate per 1000 child-years of follow-up was calculated by selected maternal and infant characteristics. The Kaplan-Meier method was used to estimate survival curves by child's HIV infection status and timing of HIV infection. Individual data from 12 studies were pooled, with 12,112 children of HIV-infected women. Mortality rates per 1,000 child-years follow-up were 39.3 and 381.6 for HIV-uninfected and infected children respectively. One year after acquisition of HIV infection, an estimated 26% postnatally and 52% perinatally infected children would have died; and 4% uninfected children by age 1 year. Mortality was independently associated with maternal death (adjusted hazard ratio 2.2, 95%CI 1.6–3.0), maternal CD4<350 cells/ml (1.4, 1.1–1.7), postnatal (3.1, 2.1–4.1) or peri-partum HIV-infection (12.4, 10.1–15.3).

**Conclusions/Results:**

These results update previous work and inform future UNAIDS modelling by providing survival estimates for HIV-infected untreated African children by timing of infection. We highlight the urgent need for the prevention of peri-partum and postnatal transmission and timely assessment of HIV infection in infants to initiate antiretroviral care and support for HIV-infected children.

## Introduction

Sub-Saharan Africa remains the region most heavily affected by HIV. In 2008, an estimated 1,800,000 HIV-infected children under 15 years were living in sub-Saharan Africa, and this continent accounted for 91% of new HIV infections among children [1]. While it is important to have accurate estimates of survival when children are treated with antiretroviral therapy, assumptions about survival of HIV-infected children in Africa in the absence of treatment [2], [3] need to be updated and refined to inform ongoing UNAIDS modelling of HIV epidemiology among children. For this purpose, UNAIDS convened a working group to attempt to pool data from all available clinical trials on mother-to-child HIV transmission prevention conducted in sub-Saharan Africa over the last 15 years, to reliably assess mortality rates in HIV-infected children.

More specifically, precise estimates of infant survival by timing of HIV-infection (perinatally or postnatally through breastfeeding) are urgently needed. Such estimates will provide benchmarks for assessing the impacts of paediatric HIV treatment whilst allowing for the effect of background mortality on survival post-infection of children. We here present the results based on data with consistent information on timing of acquisition of infection, both before or during delivery, and postnatally to reliably estimate infant survival by timing of HIV-infection. We update the previously conducted individual patient meta-analysis on survival in HIV-exposed children [3] which had limited statistical power especially on the timing of acquisition of infection.

## Methods

**Figure 1:**
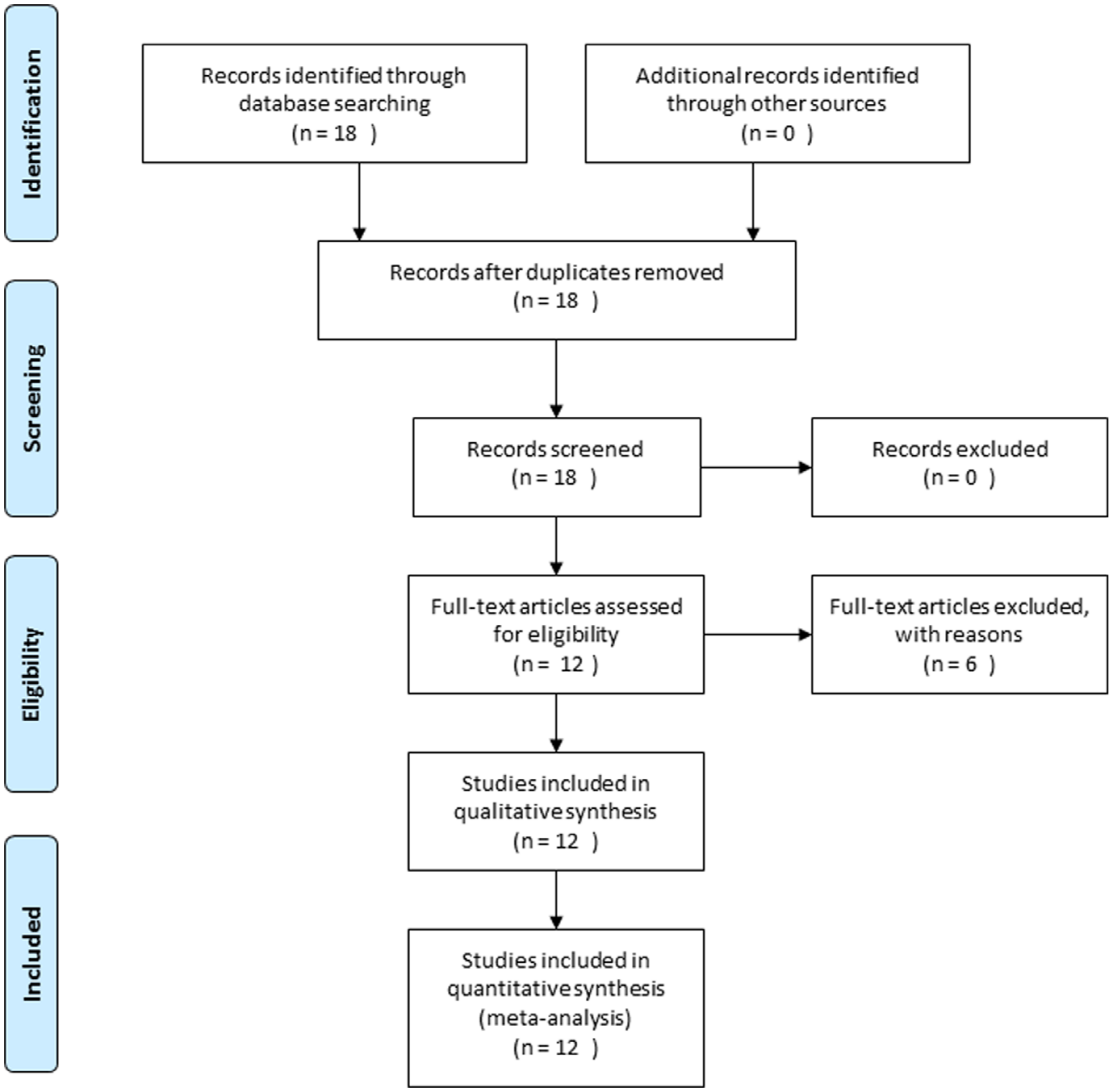
The PRISMA flow chart showing the progress of trials through the review.

Studies eligible for this pooled analysis were clinical trials or cohort studies in sub-Saharan African countries that investigated the effect of antiretroviral-based and/or infant feeding interventions in risk reduction of HIV vertical transmission with paediatric follow-up for 18–24 months and information about infant feeding. Overall, 18 trials and cohort studies aimed at the prevention of mother-to-child transmission of HIV in African settings were eligible for this pooled analysis and contacted (Figure 1). Of these, four teams did not want to participate or could not share the data at the time of the analysis [4], [5], [6], [7], two datasets were excluded (missing key variables) [8], [9], and 12 provided the necessary data [10], [11], [12], [13], [14], [15], [16], [17], [18], [19], [20], [21]. The PRISMA checklist is available as supporting information; see Checklist S1.

Paediatric HIV infection was defined as a positive plasma HIV-1 DNA/RNA PCR at any age. Children with a positive HIV-1 diagnostic test result before 4–6 weeks of age (depending on the timing of collection of blood samples in each study) were considered to be HIV-infected in peri-partum [22], [23]. This includes infants infected in utero and DNA/RNA positive on the day of birth. Children with a negative DNA/RNA PCR from a sample obtained at or after 4–6 weeks of age who subsequently acquired infection were considered to be HIV-infected postnatally [22], [23]. Children with positive test results at or after 4–6 weeks of age but with either no previous negative test result, or last negative test result before 4–6 weeks were considered infected with unknown timing of infection [22], [23].

Antiretroviral treatment became available to children in the Mashi trial on the first of October 2002, so follow-up was rightly censored at this point. Infant antiretroviral treatment was not available during the time of the other trials. Maternal antiretroviral treatment was available in the Mitra Plus study only.

Baseline characteristics were described for each study included in the pooled analysis. Overall mortality as a rate per 1000 child-years of follow-up was calculated by selected maternal and infant characteristics. The Kaplan-Meier method was used to estimate survival curves among HIV-infected children: children infected in peri-partum, infected in postnatal, and infected with unknown timing of infection. Estimated time of diagnosis of HIV-infection for those infected postnatally was taken as mid-way between the last HIV-negative test and the first HIV-positive test. The survival of children infected peri-partum or with unknown timing was estimated from birth. Studies were right-censored at the time of infant antiretroviral initiation. We used random-effects Weibull regression models to estimate mortality hazard ratios accounting for heterogeneity between trials and cohorts. Adjusted hazard ratios were obtained, both overall and for HIV-uninfected and HIV-infected children separately. The multivariable models included the following variables: region of origin (South Africa, East Africa, West Africa), sex (boys, girls, unknown), maternal vital status (time-varying: alive, dead), antenatal maternal CD4 count in cells/ml (≥350, <350, unknown), breastfeeding practice in the first year of life (ever, never), and child HIV-infection status (uninfected, determined infected in peri-partum, infected postnatally, infected with unknown timing). Age at time of infection was allowed for in the model.

## Results

The characteristics of the datasets included in the first step analysis are detailed in Table 1 and Table 2. Overall 12,112 infants born to HIV-infected mothers were included. Children with unknown HIV status were excluded from the analysis (n = 639). Analysis was therefore conducted among uninfected children (n = 8,964) and HIV-infected (n = 2,509, HIV infection acquired perinatally, through breastfeeding, or with unknown timing).

**Table 1:** Characteristics of the mother-infant pairs included in the pooled analysis (first generation studies, before year 2000).

	ANRSa Ditrame Trial, *Côte d'Ivoire, Burkina Faso* (Dabis Lancet 99)	ANRSb Ditrame Trial, *Burkina Faso, Côte d'Ivoire* (Msellati Sex Transm Infect 99)	Nairobi Trial, *Kenya* (Nduati Lancet 01)	Petra Trial, *Tanzania, South Africa, Uganda* (Petra Group Lancet 02)	Retro-CI Trial, *Côte d'Ivoire* (Wiktor Lancet 99)	Vitamin A Trial, *South Africa* (Coutsoudis Lancet 99)
Mother-infant pairs	401	104	197	1458	261	660
Follow-up (days), median (IQR)	550 (303–639)	542 (293–589)	729 (332–849)	548 (388–554)	730 (290–1008)	300 (128–456)
Maternal Age (years), mean (SD)	25.6 (5.3)	24.5 (5.0)	23.9 (4.4)	27.0 (8.1)	26.4 (5.2)	26.2 (4.7)
Antenatal maternal CD4, median (IQR)	541 (359–741)	524 (384–768)	392 (262–530)	449 (295–630)	549 (354–726)	440 (311–597)
Maternal death, n (%)	18 (4.5)	9 (8.7)	17 (8.6)	61 (4.2)	9 (3.4)	5 (0.8)
Child age at maternal death (days), median (IQR)	274 (80–561)	342 (97–379)	240 (171–413)	341 (234–441)	142 (128–319)	229 (13–232)
Male child, n (%)	207 (51.6)	49 (47.2)	96 (48.7)	726 (49.9)	131(50.2)	317 (50.3)
Birthweight<2.5 kg, n (%)	62 (15.5)	18 (17.3)	13 (6.6)	96 (6.6)	27 (10.3)	74 (11.2)
Child ever breastfed, n (%)	401 (100.0)	104 (100.0)	197 (100.0)	1397 (95.8)	261 (100.0)	452 (68.5)
Breastfeeding duration (days), median (IQR)	274 (183–295)	342 (231–484)	398 (152–548)	212 (69–420)	458 (336–559)	90 (30–278)
Child death, n (%)	88 (22.0)	26 (25.0)	45 (22.8)	184 (12.6)	39 (14.9)	49 (7.4)
Child age at death (days), median (IQR)	202 (88–429)	175 (54–505)	247 (122–366)	171 (82–293)	101 (4–339)	86 (36–153)
HIV-infected child, n (%)	89 (24.0)	14 (16.5)	59 (30.9)	253 (18.1)	68 (27.2)	143 (22.8)
*Peri-partum HIV infection, n*	*38*	*1*	*27*	*110*	*41*	*111*
*Postnatal HIVinfection, n*	*22*	*5*	*19*	*87*	*23*	*22*
*Unknown timing of HIV infection, n*	*29*	*8*	*13*	*56*	*4*	*10*

**Table 2:**
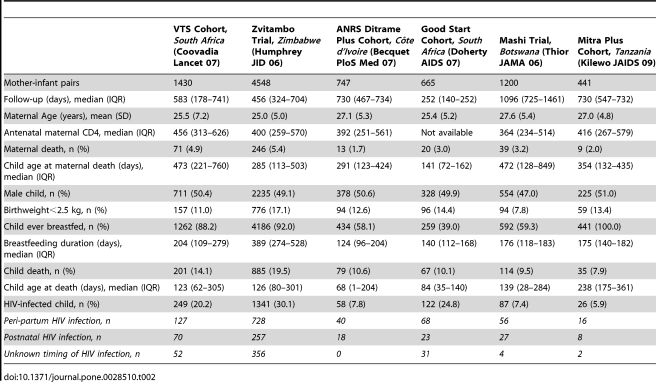
Characteristics of the mother-infant pairs included in the pooled analysis (second generation studies, after year 2000).

Overall, 1,363 children acquired infection perinatally with median time of first positive DNA/RNA test at 19 days (interquartile range: 1–42 days), 581 children were negative at or after 4 weeks of age and acquired infection through breastfeeding at a median 24 weeks of age (interquartile range: 7–39 weeks), and 565 children were infected with unknown timing of infection and had a first positive DNA/RNA test at 80 days in median (interquartile range: 51–168 days).

Crude mortality rates per 1,000 child-years of follow-up were 39.3 and 381.6 for HIV-uninfected and HIV-infected children, respectively. Mortality rate per 1000 child-years of follow-up by selected maternal and infant characteristics are detailed in Table 3.

**Table 3:** Mortality rate per 1000 child-years of follow-up by selected maternal and infant characteristics (n = 12,112).

	Number (n = 12,112)	Deaths (n = 1,812)	Mortality rate per 1000 child-years of follow-up
**Location**			
Southern Africa	9270 (76.5)	1384	105.8
Eastern Africa	1329 (11.0)	196	100.1
Western Africa	1513 (12.5)	232	100.0
**Maternal PMTCT ARV**			
None	6634 (54.8)	1179	149.2
sdNVP	1657 (13.7)	223	99.3
2 or 3 drugs regimen	3821 (31.5)	410	56.8
**Infant PMTCT ARV**			
None	6892 (46.9)	1227	141.9
sdNVP +/− ZDV/3TC	5220 (53.1)	585	67.1
**Maternal CD4 count**			
<350	3973 (32.8)	796	135.2
≥350	6605 (54.5)	776	77.2
Unknown	1534 (12.7)	240	167.7
**Maternal vital status**			
Mothers known alive	11595 (95.7)	1598	95.6
Mothers known dead	517 (4.3)	214	332.3
**Sex of the infant**			
Girls	5957 (49.2)	883	103.5
Boys	6072 (50.1)	918	104.3
Unknown	83 (0.7)	11	-
**Infant birthweight**			
<2.5 kg	1566 (12.9)	445	232.4
≥2.5 kg	10372 (85.6)	1333	87.3
Unknown	174 (1.5)	34	-
**Breastfeeding status**			
Never	2126 (17.5)	310	113.9
Ever	9986 (82.5)	1502	102.6
**Child infection status**			
HIV-uninfected	8964 (74.0)	562	39.3
HIV-infected	2509 (20.7)	1096	381.6
Unknown	639 (5.3)	154	-

**Figure 2:**
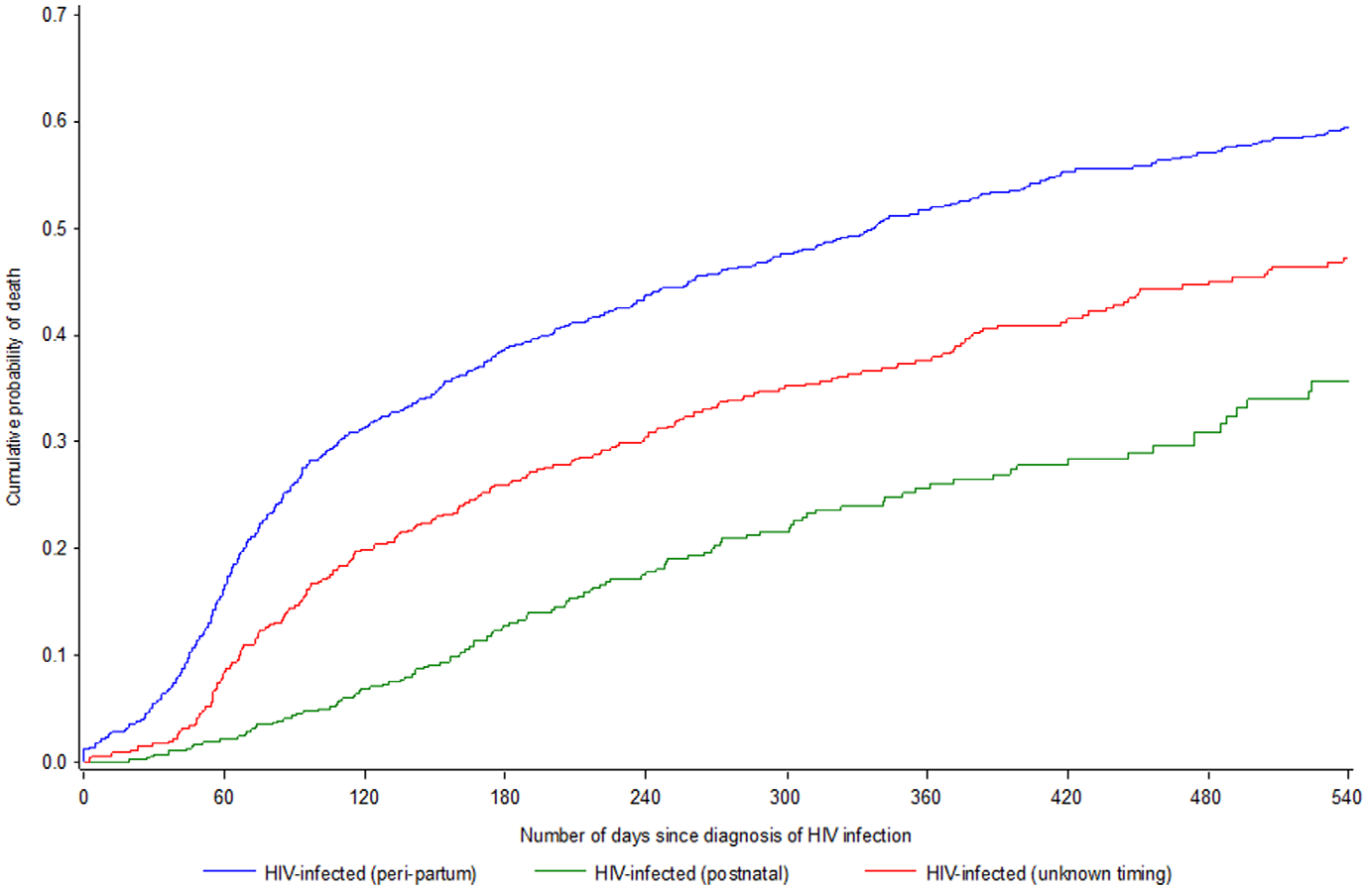
Estimated 18-month unadjusted mortality for HIV-infected children since acquisition of HIV infection (n = 2,509).

Mortality was estimated for HIV-infected children by timing of transmission; initial time was the estimated date of acquisition of HIV-infection (Figure 2). Overall, 12 months post-acquisition of infection, an estimated 52% of children with peripartum infection (95% confidence interval [CI]: 49–55%) and 26% (95% CI: 22–31%) of those with postnatal infection died. Among children with unknown timing, an estimated 38% (95% CI: 22–31%) died compared with an estimated 4% of uninfected children who died by age 1 year. Detailed probabilities are available as supporting information; see Table S1.

Adjusted mortality hazard ratios (aHR) with 95% CI by selected characteristics were estimated in regression analyses among HIV-infected and uninfected children (n = 11,473). With South Africa as reference, mortality was significantly higher in East Africa, aHR 1.41 (1.14–1.74), but not West Africa, aHR 1.20 (0.88–1.63). Mortality did not differ significantly by sex: with boys as reference, aHR in girls was 1.02 (0.76–1.36). When the mother died during follow-up, children were more than twice likely to die (aHR 2.21 (1.63–3.00)) than when mothers survived. With mothers with CD4≥350 cells/ml as reference, children born to women with CD4<350 (aHR 1.36 (1.08–1.71)) or to women with unknown CD4 (aHR 1.30 (0.72–2.37)) were at significantly increased risk of dying. Children who had never been breastfed were at more than double the risk of dying than ever breastfed children (aHR 2.21 (1.62–3.01)). Mortality was substantially and significantly associated with child HIV infection status: compared to uninfected children, the aHRs were 12.45 (10.15–15.27) for perinatally infected children, 3.08 (2.29–4.14) for children infected through breastfeeding and 7.21 (5.53–9.39) for children with unknown timing of infection.

## Discussion

Mortality of children born to HIV-infected mothers was assessed on a large dataset from three different regions in sub-Saharan Africa. Overall mortality of HIV-infected children was dramatically elevated, irrespective of timing of acquisition of infection. However, among HIV-infected children, survival from acquisition of infection in postnatally infected children was higher than in those with infection acquired around delivery: the 18-month post-infection mortality risks were 36% (30%–42%) and 60% (57%–63%), respectively. Most these children had been included in research studies offering relatively high standard of care compared to less supported field areas; a closed clinical follow-up adapted to the child's age with free provision of care was provided to these children. Hence, we assume the survival estimates presented here were higher than what would be expected in more operational field settings. Our results are in accordance with previous findings of high mortality in vertically-infected children [24]: more than half of these children will have died by age two years. While the previously conducted meta-analysis on the subject had limited statistical power to assess infant mortality according to the timing of acquisition of HIV infection, we here present more reliable estimates on this matter.

Our analysis strategy implied that the survival was assessed from the estimated timing of infection in order to assess survival after infection for perinatally and postnatally infected children. This estimate would thus need to be interpreted in the light of the selection that occurs with children at risk of postnatal infection by definition being those who survived without infection the neonatal period which is one of high mortality risk. On the other hand, perinatally infected children will have to survive this risky period with the infection as an added exposure. However, there is no evidence of an increased neonatal mortality risk associated with perinatal infection [25]. Further, complementary analyses on the same dataset showed that differences seen in the survival of perinatally or postnatally infected children cannot be explained by differences in background mortality, which is much higher in the neonatal period [26]. We suggest that our findings are explained by the immunological immaturity, and thus reduced virological control, of the fetus and new-born at the time of acquisition of infection whereas postnatal acquisition of infection in infants who are more immunologically mature would be associated with a greater chance of survival [27].

We further also confirm the impact of both maternal health, as measured by her HIV immunological progression, and vital status on infant survival. Two-year infant mortality adjusted risk was one third higher among women with ante-partum CD4 count <350 cells/ml than among those with mothers a CD4 count above this threshold; and it was two times higher when the mother had died during the two years post-partum than when the mother was alive two years after birth. Lifelong antiretroviral treatment was not available to women followed-up in all studies selected for this pooled analysis but one. By lack of statistical power, it was therefore not possible to assess the impact of maternal antiretroviral treatment on the survival of children. Bearing in mind that maternal health is an important confounder of child survival, it would be crucial to investigate whether there is a positive impact of such a maternal treatment on infant survival. As maternal antiretroviral treatment keeps an infected mother alive, there would be an indirect effect of reducing child mortality in both her HIV-infected and uninfected children.

We present estimates of risk of mortality in children born to HIV-infected mothers in Africa, on the basis of a large dataset with wide variations in the characteristics of the patients included in the meta-analysis. The individual meta-analysis and adjustments in the model account for some of these effects. We acknowledge that there may have been confounding factors that had not been taken into account by our design, but we consider that the most important ones were controlled for. Additionally, the science in the prevention of mother-to-child transmission of HIV has changed since the time of the studies included in this meta-analysis. Expanding the indication of use of potent antiretroviral drug combinations to all pregnant, delivering, and breast-feeding HIV-infected women is now seen as an unprecedented opportunity to radically reduce the burden of paediatric AIDS [28]. The implication of this on the predicted trends in child mortality will need to be carefully assessed in future studies.

Our findings draw attention to the importance of comprehensive care for HIV-infected women. More specifically, our results stress the urgency of providing optimal antiretroviral treatment to women who need it for their own health (e.g. with ante-partum CD4 count <350 cell/ml according to the newly revised WHO guidelines [29]) to also improve survival of their (infected and uninfected) children. Maternal antiretroviral treatment will radically reduce the burden of paediatric HIV worldwide, and contribute to the improvement of survival of children born to HIV-infected women in improving maternal health and considerably lowering the risk of maternal death [30]. Moreover, there is still an urgent need for the effective prevention of mother-to-child transmission of HIV through breastfeeding: even postnatally infected children are three-times more likely to die than uninfected children. These findings also highlight the urgent need for the early assessment of HIV infection in HIV-exposed children to allow the timely initiation of antiretroviral care and support for HIV-infected children. Finally, these revised estimates of infant survival by timing of acquisition of HIV infection will now be used by UNAIDS to improve forecasts of the future of the HIV pandemic [31].

## Supporting Information

Checklist S1 **PRISMA checklist.**
(DOC)

Table S1 **Probability with 95%CI of 18-month unadjusted mortality for HIV-infected children since acquisition of HIV infection (n = 2,509).**
(DOCX)
