# Supplementary material for: Children Who Acquire HIV Infection Perinatally Are at Higher Risk of Early Death than Those Acquiring Infection through Breastmilk: A Meta-Analysis
Source: PLoS One. 2012 Feb 23;7(2):e28510. doi: 10.1371/journal.pone.0028510 (PMC3285615; doi:10.1371/journal.pone.0028510)
Supplement: Table S1 — Probability with 95%CI of 18-month unadjusted mortality for HIV-infected children since acquisition of HIV infection (n = 2,509). (DOCX) [file pone.0028510.s002.docx]

|  | **90 days** | **180 days** | **365 days** | **540 days** |
| --- | --- | --- | --- | --- |
| **Peri-partum HIV infection, n=1,363** |  |  |  |  |
| Children at risk | 912 | 740 | 461 | 240 |
| Death probability | 0.26 | 0.39 | 0.52 | 0.60 |
| 95% CI | 0.24-0.29 | 0.36-0.42 | 0.49-0.55 | 0.57-0.63 |
| **Postnatal HIV infection, n=581** |  |  |  |  |
| Children at risk | 458 | 351 | 170 | 71 |
| Death probability | 0.04 | 0.13 | 0.26 | 0.36 |
| 95% CI | 0.03-0.06 | 0.10-0.16 | 0.22-0.31 | 0.30-0.42 |
| **Unknown timing HIV infection, n=565** |  |  |  |  |
| Children at risk | 466 | 368 | 229 | 127 |
| Death probability | 0.15 | 0.26 | 0.38 | 0.47 |
| 95% CI | 0.12-0.18 | 0.22-0.30 | 0.34-0.42 | 0.43-0.52 |
